# Supplementary material for: Efflux pump activity potentiates the evolution of antibiotic resistance across S. aureus isolates
Source: Nat Commun. 2020 Aug 7;11:3970. doi: 10.1038/s41467-020-17735-y (PMC7414891; doi:10.1038/s41467-020-17735-y)
Supplement: Supplementary file 1 — Supplementary Information [file 41467_2020_17735_MOESM1_ESM.pdf]

Supplementary Information

Papkou et al. Efflux pump activity potentiates the evolution of antibiotic resistance across S. aureus isolates

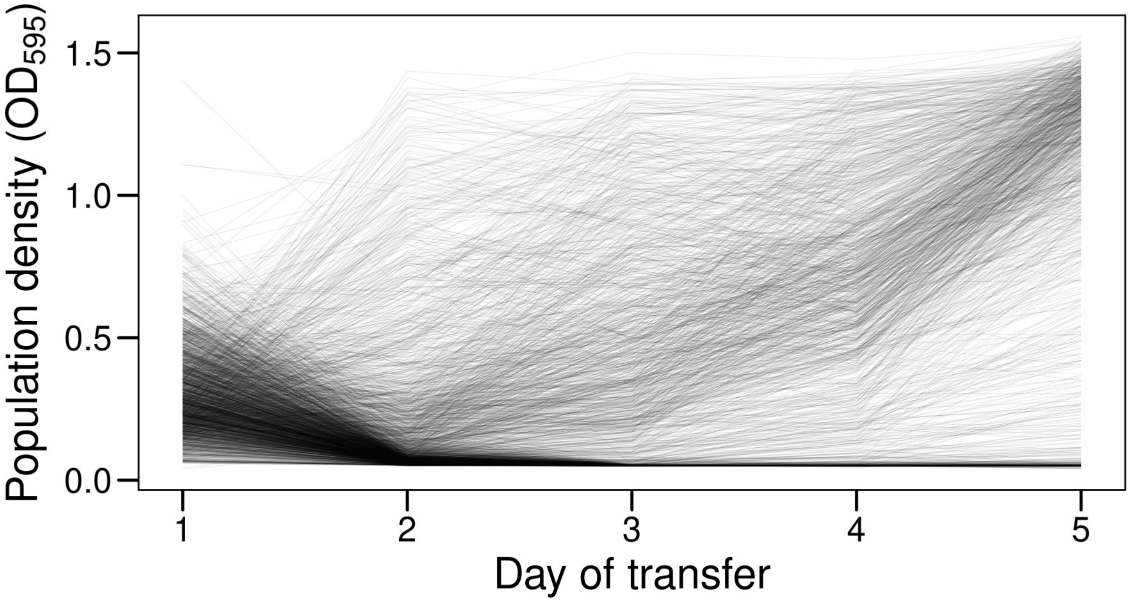

Supplementary Fig. 1.

Population density during experimental evolution (*N*=2664). After 5 transfers, 1075 out of 2664 populations had detectable bacterial growth (optical density at λ=595 > 0.08). 1589/2664 populations went extinct

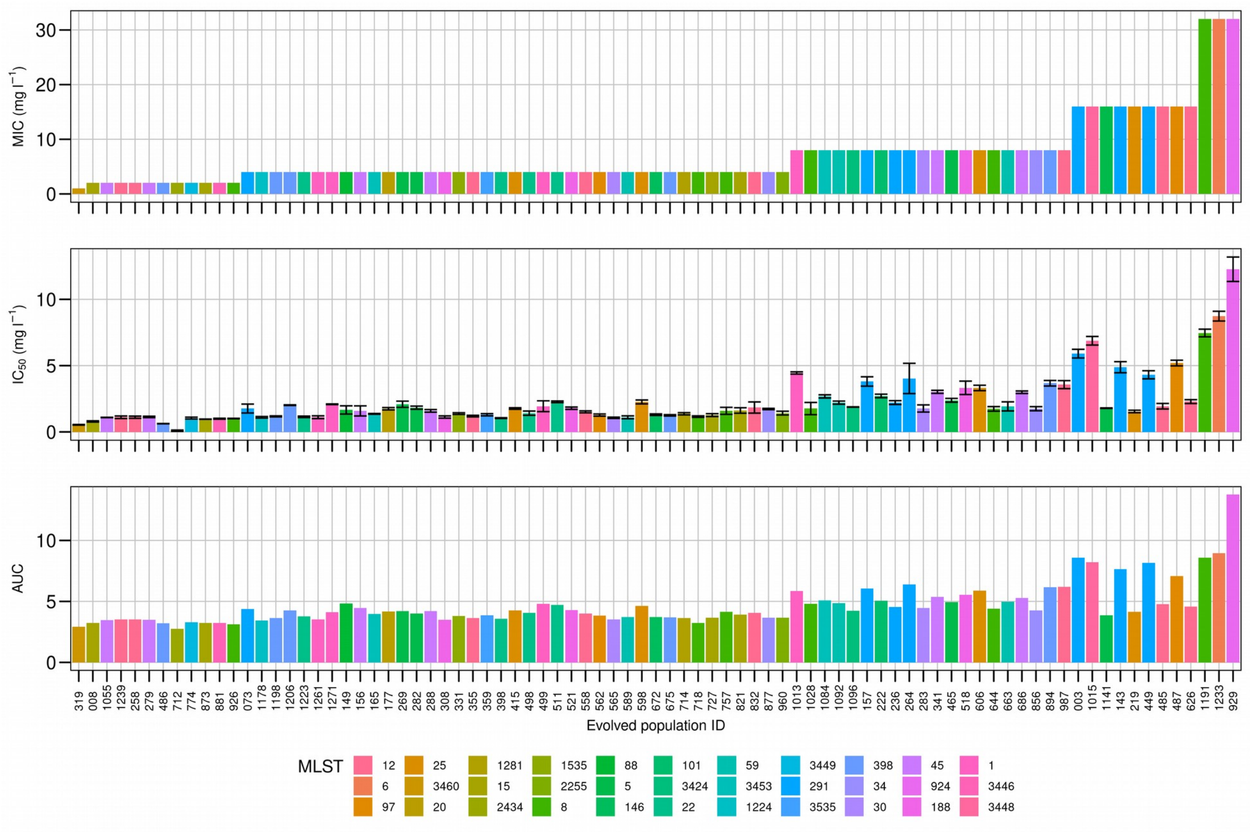

Supplementary Fig. 2.

Resistance in the subset of the evolved populations. Resistance was measured as minimal inhibitory concentration (MIC), half growth inhibition dose (IC$_{50}$) or area under a dose-response curve (AUC), N=83. Each population was tested at 8 doses of ciprofloxacin using 5 replicate cultures per dose. MIC was calculated as a minimal dose at which no growth was observed in 3 out of 5 replicates. IC$_{50}$ was estimated as a parameter by fitting a dose-response model using the drc package in R. The standard errors for IC$_{50}$ were calculated using the same model (shown as error bars). Colours indicate multilocus sequence type (MLST) of populations

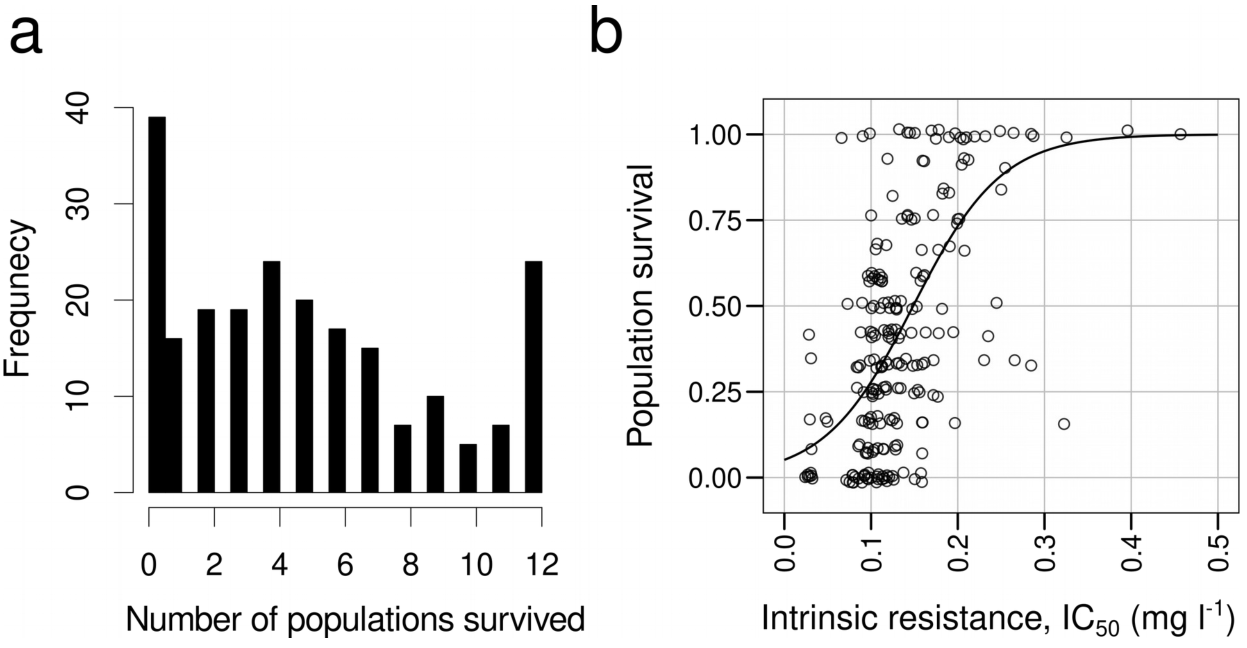

Supplementary Fig. 3.

The distribution of the number of surviving populations. a The distribution of the number of surviving populations (*N*=222). **b** The effect of intrinsic resistance on evolvability (*N*=222). Solid line shows survival probability as predicted by generalized liner model ($\chi^2 = 358.77$, *d.f.*=1, $p< 2.2e\text{-}16$, also see Supplementary Table 1)

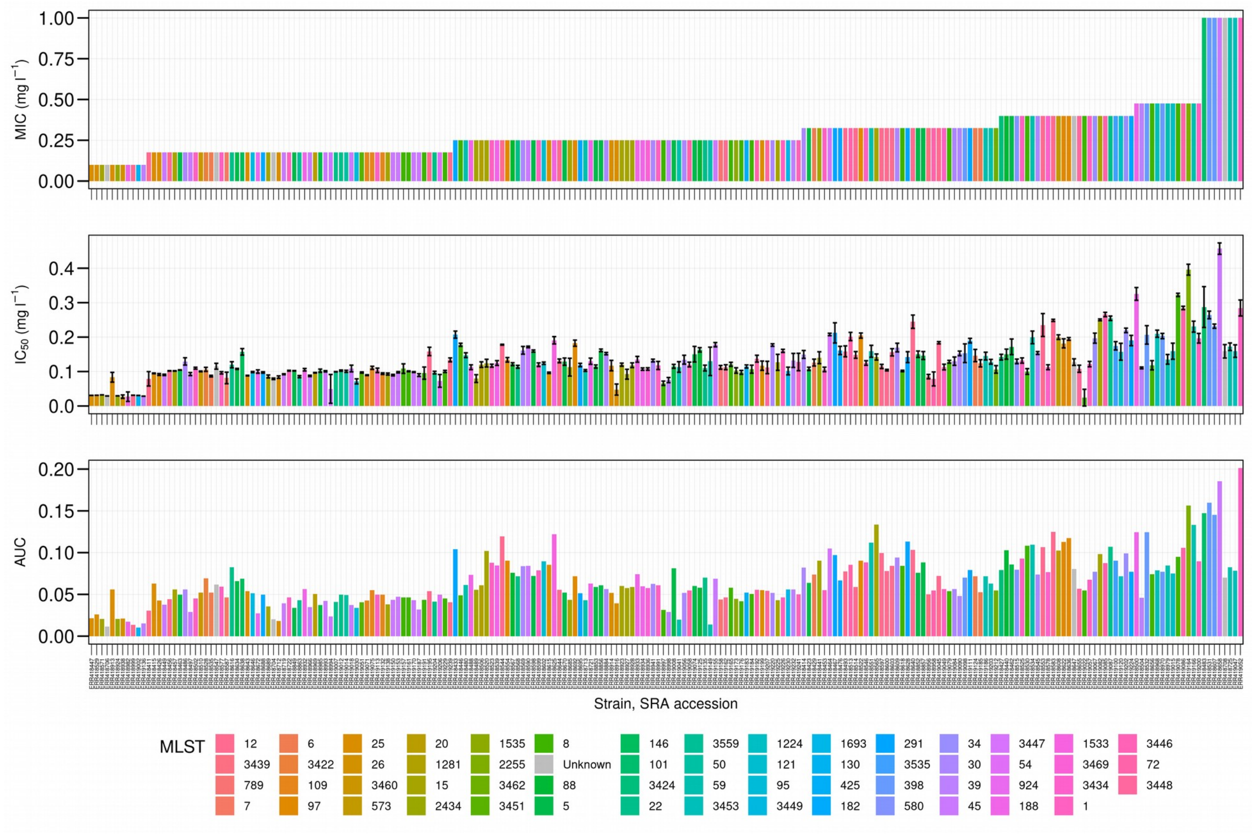

Supplementary Fig. 4.

Initial intrinsic resistance of 222 parental strains. Resistance was measured as minimal inhibitory concentration (MIC), half growth inhibition dose (IC_{50}) or area under a dose-response curve (AUC). Each strain was tested at 8 doses of ciprofloxacin using 5 replicate cultures per dose. MIC was calculated as a minimal dose at which no growth was observed in 3 out of 5 replicates. IC_{50} was estimated as a parameter by fitting a dose-response model using the drc package in R. The standard errors for IC_{50} were obtained using the same model (shown as error bars). Colours indicate multilocus sequence type (MLST) of strains.

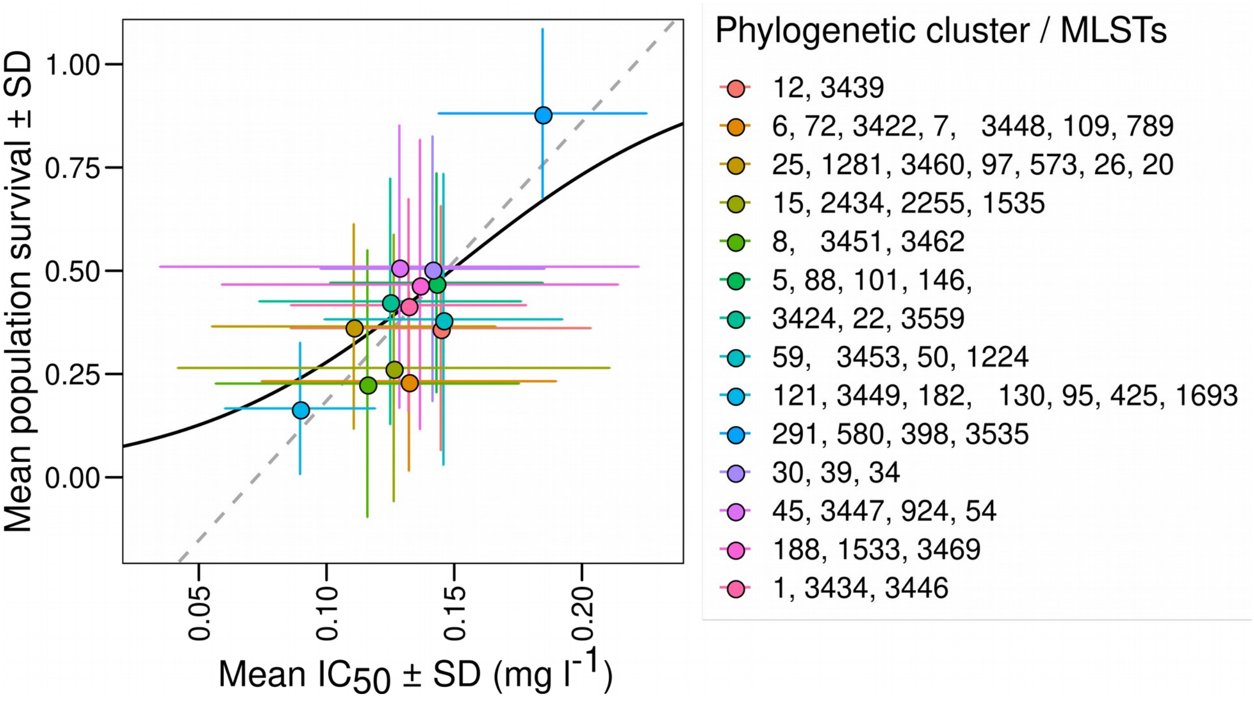

Supplementary Fig. 5.
Correlation between evolvability and intrinsic resistance at the level of phylogenetic clusters. Data points show mean evolvability (y-axis) and mean intrinsic resistance (initial half growth inhibition dose IC_{50}, x-axis) averaged for 14 phylogenetic clusters. Error bars represent standard deviations. The solid black line shows the population survival probability modelled for all parental strains using logistic regression (Supplementary Table 1). The dashed line depicts a simple correlation based on 14 data points representing means for phylogenetic clusters (Pearson's correlation r=0.825, t = 5.06, n = 14, p < 0.0003). The legend on the left shows *S. aureus* multilocus sequence types (MLST) within in each cluster. The number of strains used for calculating a mean and standard deviation was: cluster 1 =18, cluster 2 =19, cluster 3 =21, cluster 4 =17, cluster 5 =18, cluster 6 =20, cluster 7 =9, cluster 8=17, cluster 9 =8, cluster 10 =14, cluster 11 =17, cluster 12 =17, cluster 13 =10, and cluster 14 =17)

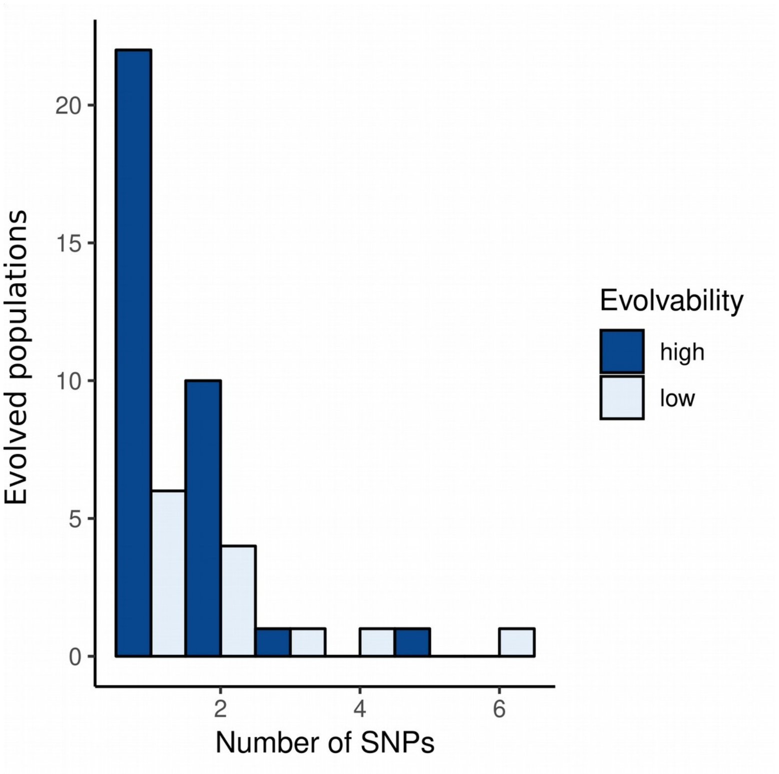

Supplementary Fig. 6.

The distribution of a number of SNPs in evolved populations. The distribution is compared between populations from high evolvability (evolvability >=9/12, dark blue) and low evolvability (evolvability <=2/12, light blue) parental strains. The number of strains shown is *N*=13 for low evolvability strains and *N*=34 for high evolvability strains

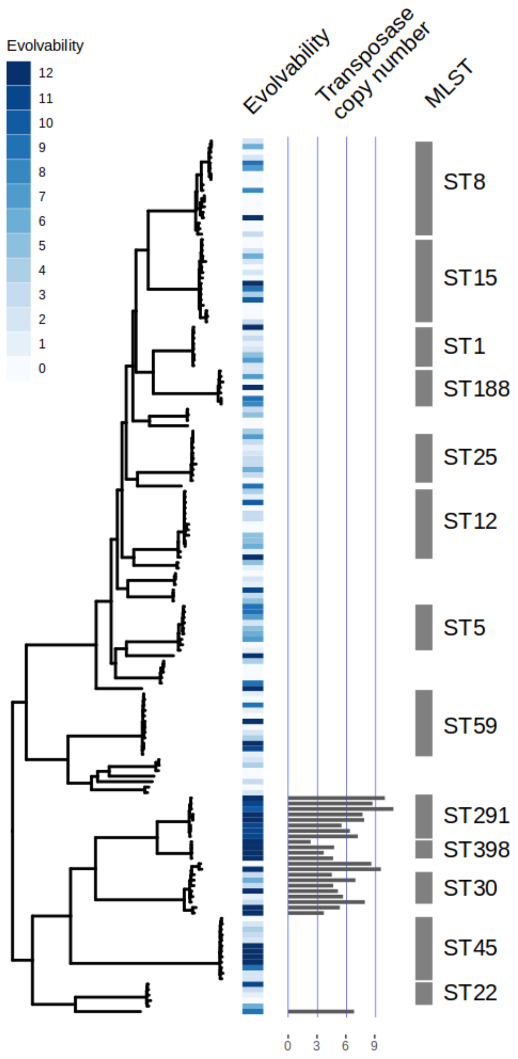

Supplementary Fig. 7.

Maximum likelihood phylogenetic tree of parental strains. The tree is annotated with evolvability (low = light blue, high = dark blue) and estimated copy number of the transposase JP02758_0628/0637. Sequence data were mapped to the JP02758_0628 gene in the JP80 reference genome using Stampy. Copy number was calculated as the mean sequencing depth across the transposase, normalized by mean sequencing depth across the genome from mapping to the MRSA252 reference sequence

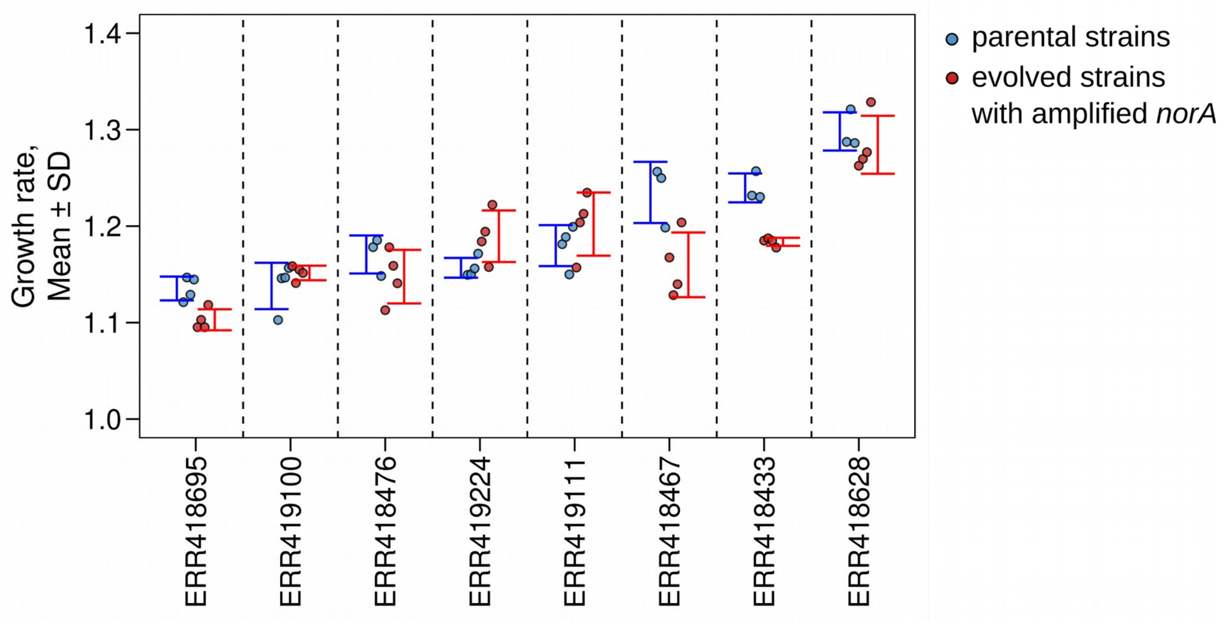

Supplementary Fig. 8.

The effect of *norA* amplification on growth rate in the absence of ciprofloxacin. Maximum growth rate is shown for parental strains (blue circles) and the evolved populations from clade CC398 that amplified *norA* (red circles). The growth rate was estimated as a maximum slope of growth curves during log phase in the MH2 medium without ciprofloxacin. The error bars show standard deviation of growth rate estimates in independent cultures. The number of independent cultures for each strain was the following: n=3 for a parental population and n=4 for an evolved population in strains ERR418433, ERR418467, ERR418476, ERR418628; n=4 for a parental population and n=4 for an evolved population in strains ERR418695, ERR419100, ERR419111, ERR419224. Wilcoxon signed rank test (paired, two-sided) comparing the means for ancestral and evolved populations: $W = 26$, $N = 8$, $p = 0.3125$

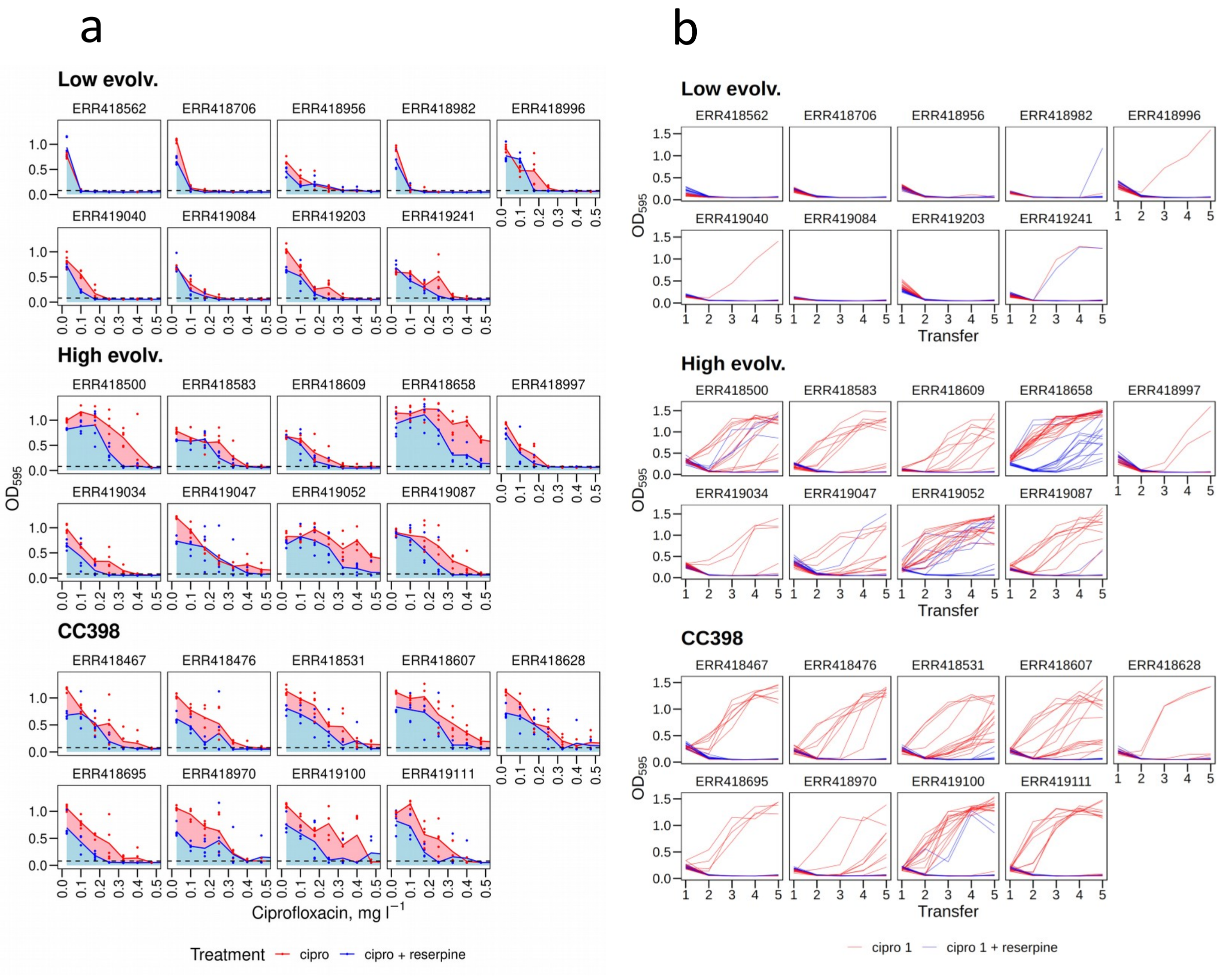

Supplementary Fig. 9.

The effect of reserpine on ciprofloxacin resistance and evolvability. a The effect of the efflux pump inhibitor (reserpine, 33 µM) on *S. aureus* resistance to ciprofloxacin. 27 strains representing high and low evolvability isolates as well as isolates from CC398 were exposed to a range of ciprofloxacin concentrations with (blue) or without of inhibition (red). Red and blue lines show the means of optical density calculated using 5 replicates per strain/treatment/concentration. For statistical analysis, see Supplementary Table 11. **b** The effect of reserpine on evolvability. The same 27 strains were evolved to ciprofloxacin (1 mg l^{-1}) for 5 transfers with reserpine (blue lines) or without reserpine (red lines). *N*=16 replicate populations were included per each strain/treatment combination. For statistical analysis, see Supplementary Table 12

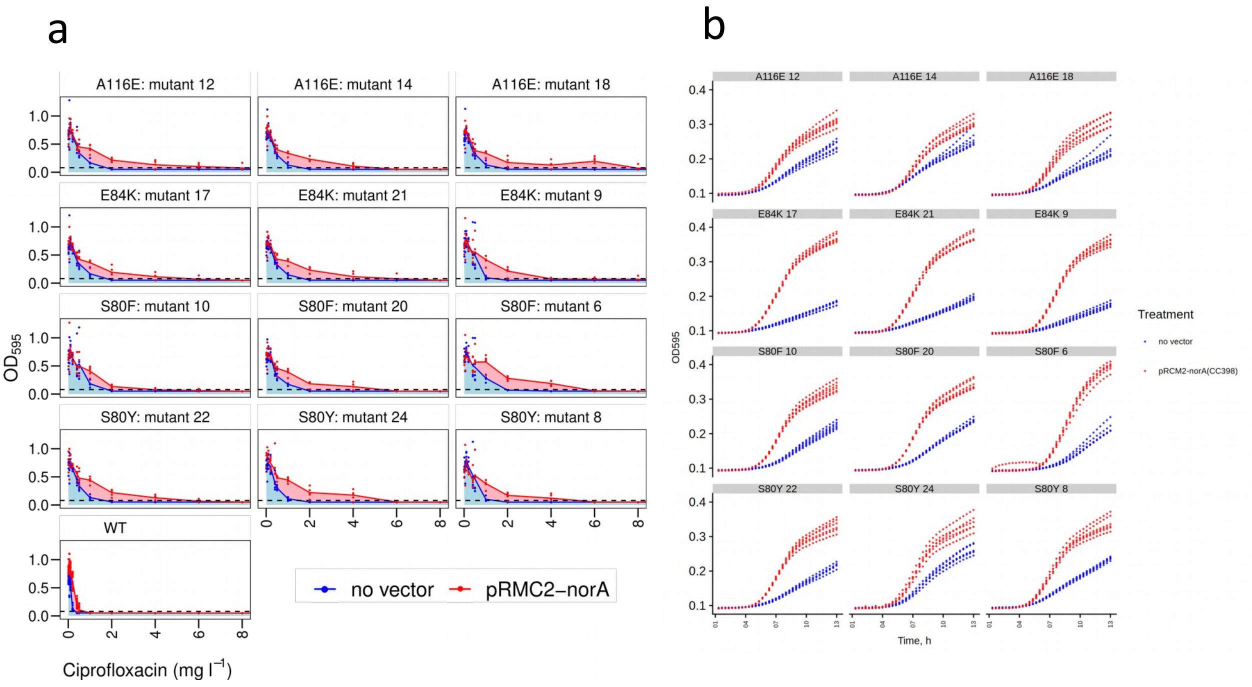

Supplementary Fig. 10.

The effect of *norA* expression on ciprofloxacin resistance and growth. a The effect of *norA* expression on the resistance of *grlA* mutants to ciprofloxacin. The single-point mutants were obtained by plating independent cultures of the sensitive RN4220 strain on agar plates with ciprofloxacin. The mutants were verified by sequencing and transformed with pRMC2-*norA* vector. For determining resistance, the mutants were exposed to 13 different concentrations of ciprofloxacin (0.01-12 mg l^{-1}) using 5 replicate cultures per concentration. Read lines show mean optical density for mutants carrying pRMC2-*norA*, and blue line show density for mutants without vector. **b** The growth curves of *grlA* mutants resistant to ciprofloxacin at 1 mg l^{-1} of the antibiotic depending on the presence of pRMC2-*norA* vector (pRMC2-*norA* = red, no vector = blue). Each mutant was obtained and transformed independently and tested using 6 replicates cultures

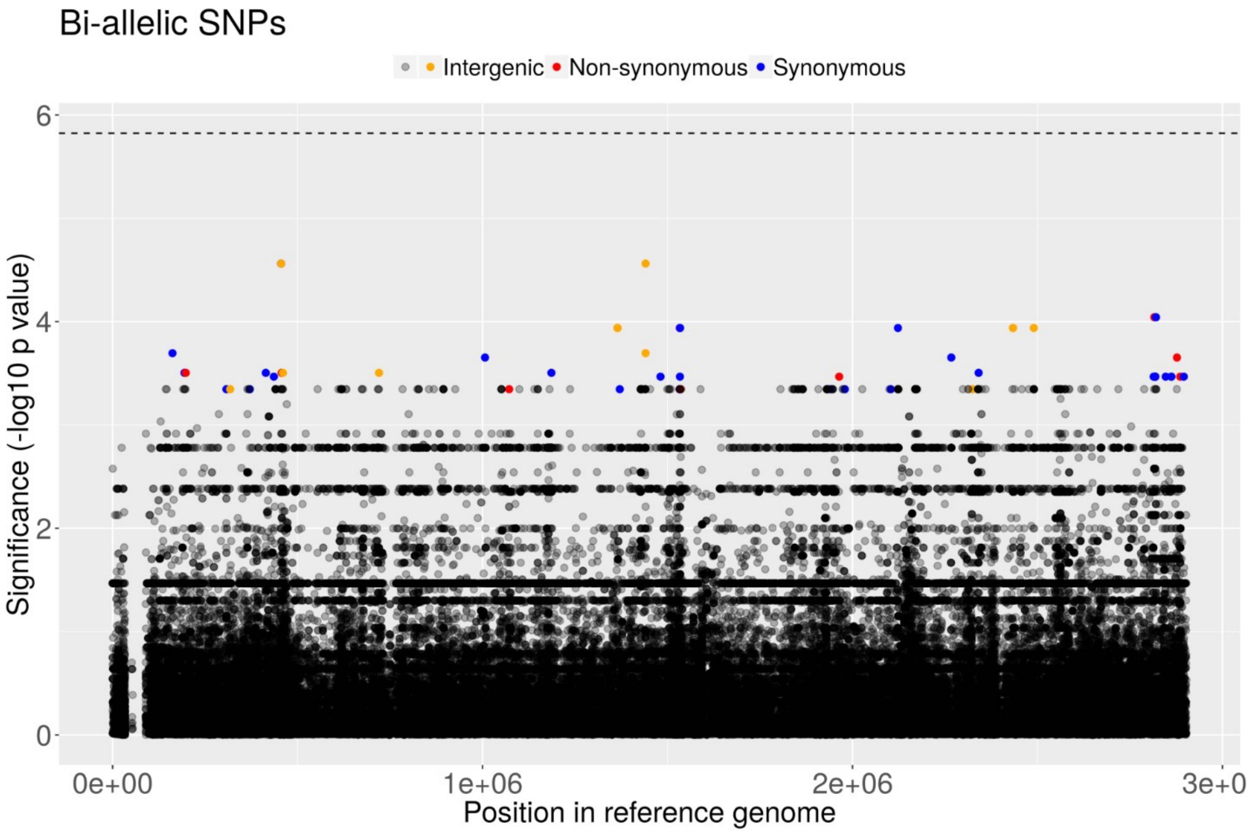

Supplementary Fig. 11.

Genome-wide association analysis of *S. aureus* evolvability to ciprofloxacin. Significance of association with evolvability for all bi-allelic SNPs called against the MRSA252 reference genome. The top 0.1% of SNPs are coloured by the SNP effect: non-synonymous = red, synonymous = blue, intergenic = gold. The dashed line shows the Bonferroni-corrected significance threshold

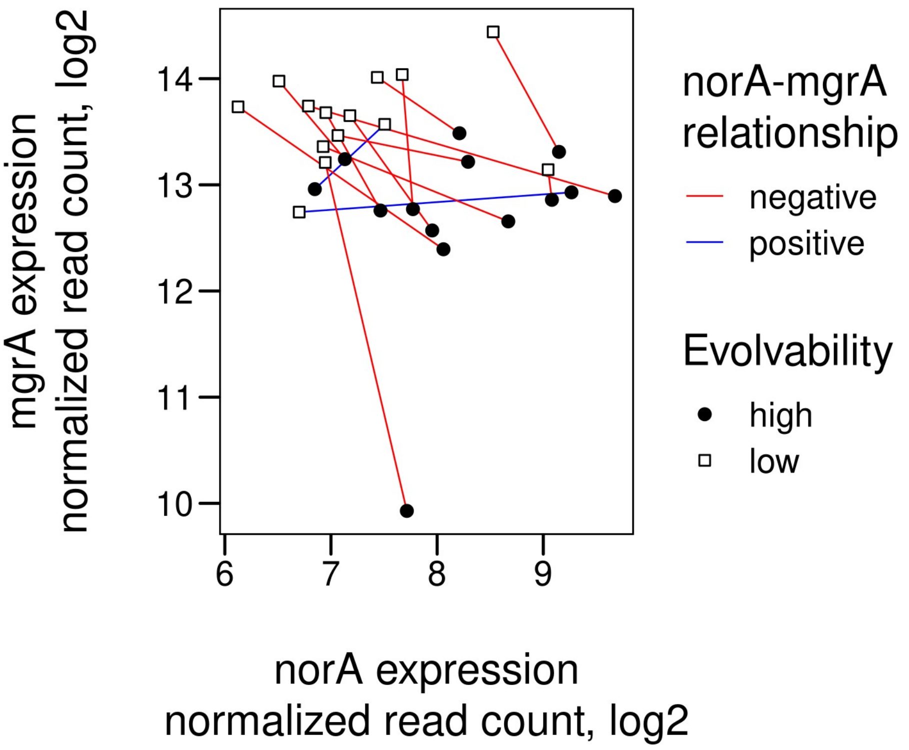

Supplementary Fig. 12.

The correlation between gene expression of *norA* and *mgrA*. The normalized read counts of *norA* and *mgrA* genes are shown for a set of 28 parental strains. The pairs of high and low evolvability strains are depicted as dots connected by lines (squared open dots = low evolvability strains, filled circular dots = high evolvability strains). Different line colours indicate positive or negative relationship between the expression of *norA* and *mgrA* within these pairs: red lines = negative relationship, blue lines = positive relationship. Spearman's rank correlation: $\rho = -0.3426382$, $N = 14$, $p = 0.07484$.

Supplementary Table 1.

Regression analysis of intrinsic resistance as a predictor of evolvability. The deviance table is shown [1a]

	Df	Deviance	Resid. Df	Resid. Dev	% variation explained[b]	Pr(>Chi)	
IC_{50}	1	393.928	220	1063.227	27.03	< 2.2e-16	***
Cluster	13	187.126	207	876.101	39.88	< 2.2e-16	***
IC_{50} × Cluster	13	55.416	194	820.685	41.06	3.415e-07	***

Null deviance =1457.16, *d. f.*=221

[a] – We fitted a generalized linear model (binomial distribution of errors) using the number of survived/extinct replicates as a response variable. The effect of intrinsic resistance (IC_{50}, half growth inhibition dose) was considered as a continuous covariate. To account for the effect of phylogeny, the isolates were clustered into 14 groups (with 8-21 isolate per cluster) based on phylogenetic distance and, then, the belonging to one of these clusters was used as a fixed predictor. The analysis was performed using R (version 3.5.1).

[b] – The proportion of variation in evolvability explained by model's terms. The terms were added sequentially, from the null model to the full model. The proportion of explained variance was calculated as 1- (Residual deviance/Null deviance) (see ref. 1 p. 383).

Supplementary Table 2

Summary of fitted coefficients for the regression exploring the relationship between intrinsic resistance and evolvability [c]

Term	Estimate	Std. Error	Two-sided Wald test		
			z value	p-value	
Intercept	-2.929	0.483	-6.069	1.29e-09	***
IC50	15.686	2.969	5.283	1.27e-07	***
cluster2	0.466	0.628	0.741	0.45841	
cluster3	0.551	0.603	0.915	0.36025	
cluster4	-1.398	0.821	-1.703	0.08853	.
cluster5	1.653	0.604	2.739	0.00616	**
cluster6	0.970	0.745	1.302	0.19286	
cluster7	-0.463	1.062	-0.436	0.66297	
cluster8	-0.081	0.795	-0.102	0.91860	
cluster9	-4.731	2.223	-2.128	0.03332	*
cluster10	0.415	1.322	0.313	0.75392	
cluster11	0.650	0.772	0.842	0.39961	
cluster12	0.993	0.670	1.482	0.13840	
cluster13	0.219	0.842	0.261	0.79443	
cluster14	0.999	0.701	1.425	0.15416	
IC50 × cluster2	-6.555	3.917	-1.674	0.09419	.
IC50 × cluster3	0.078	4.068	0.019	0.98479	
IC50 × cluster4	10.011	6.036	1.659	0.09720	.
IC50 × cluster5	-15.259	4.063	-3.755	0.00017	***
IC50 × cluster6	-2.725	4.930	-0.553	0.58049	
IC50 × cluster7	10.009	8.659	1.156	0.24770	
IC50 × cluster8	1.209	4.983	0.243	0.80837	
IC50 × cluster9	44.560	20.292	2.196	0.02809	*
IC50 × cluster10	10.813	8.175	1.323	0.18596	
IC50 × cluster11	0.457	5.027	0.091	0.92762	
IC50 × cluster12	1.690	5.155	0.328	0.74300	
IC50 × cluster13	4.108	6.114	0.672	0.50165	
IC50 × cluster14	-3.585	4.798	-0.747	0.45499	

[c] – The model coefficients are from the model presented in Supplementary Table 1. The coefficients and standard errors were obtained using the summary.glm function from the stats package in R (version 3.5.1). The same function was used to perform two-sided Wald test. The reported p-values were not adjusted (for post-hoc tests see Supplementary Table 5). The coefficients for cluster1 and IC50 × cluster1 are missing because they are included into intercept, as by default using treatment contrasts function in R.

Supplementary Table 3

Summary statistics of intrinsic resistance and evolvability shown for most represented MLSTs [d]

MLST	Cluster	IC_{50}, mg l^{-1}		Population survival		*N*	Pearson correlation	
		Mean	S.D.	Mean	S.D.		*r*	*p*-value
12	1	0.148	0.059	0.377	0.298	17	0.617	0.00828
72	2	0.181	0.083	0.306	0.282	6	0.447	0.37366
25	3	0.080	0.031	0.273	0.145	11	0.254	0.45087
15	4	0.116	0.048	0.202	0.281	14	0.806	0.00049
8	5	0.118	0.065	0.272	0.338	15	-0.009	0.97483
5	6	0.130	0.025	0.399	0.229	14	0.027	0.92576
22	7	0.128	0.056	0.393	0.314	7	0.767	0.04411
59	8	0.151	0.038	0.356	0.301	11	0.393	0.23158
291	10	0.174	0.035	0.929	0.058	7	-0.228	0.62254
30	11	0.149	0.016	0.424	0.243	11	0.245	0.46833
45	12	0.129	0.100	0.524	0.366	14	0.517	0.05808
188	13	0.133	0.085	0.479	0.375	8	0.651	0.08044
1	14	0.134	0.049	0.428	0.258	15	0.534	0.04055

[d] – Only MLSTs with 6 or more parental strains are shown.

Supplementary Table 4.
The correlation between intrinsic resistance to ciprofloxacin and evolvability within 14 phylogenetic clusters

Cluster	MLSTs	IC50, mg l^{-1}		Population survival		N	Pearson correlation	
		Mean	S.D.	Mean	S.D.		r	p-value
**1**	12, 3439	0.145	0.059	0.361	0.297	18	0.640	0.00424
**2**	6, 72, 3422, 7, 3448, 109, 789	0.132	0.058	0.232	0.218	19	0.495	0.03111
**3**	25, 1281, 3460, 97, 573, 26, 20	0.111	0.055	0.365	0.249	21	0.748	0.00010
**4**	15, 2434, 2255, 1535	0.126	0.084	0.265	0.324	17	0.805	0.00010
**5**	8, 3451, 3462	0.116	0.059	0.227	0.324	18	0.016	0.95027
**6**	5, 88, 101, 146,	0.143	0.042	0.471	0.267	20	0.441	0.05143
**7**	3424, 22, 3559	0.125	0.051	0.426	0.299	9	0.735	0.02419
**8**	59, 3453, 50, 1224	0.146	0.047	0.382	0.354	17	0.443	0.07482
**9**	121, 3449, 182, 130, 95, 425, 1693	0.090	0.029	0.167	0.161	8	0.815	0.01361
**10**	291, 580, 398, 3535	0.185	0.041	0.881	0.206	14	0.466	0.09306
**11**	30, 39, 34	0.141	0.044	0.505	0.322	17	0.478	0.05256
**12**	45, 3447, 924, 54	0.129	0.094	0.510	0.343	17	0.523	0.03116
**13**	188, 1533, 3469	0.137	0.078	0.467	0.352	10	0.668	0.03492
**14**	1, 3434, 3446	0.132	0.046	0.417	0.259	17	0.486	0.04801

Supplementary Table 5.

Post-hoc tests for the effect of phylogeny on the relationship between intrinsic resistance and evolvability [e]

Cluster	MLSTs	Estimate	SE	Two-sided Wald test		
				Z ratio	*p* value	
1	12, 3439	-0.540	0.167	-3.236	0.01700	*
2	6, 72, 3422, 7, 3448, 109, 789	-0.950	0.166	-5.725	1.45e-07	***
3	25, 1281, 3460, 97, 573, 26, 20	0.022	0.151	0.143	1.00000	
4	15, 2434, 2255, 1535	-0.602	0.205	-2.940	0.04588	*
5	8, 3451, 3462	-0.924	0.170	-5.438	7.56e-07	***
6	5, 88, 101, 146,	0.066	0.142	0.468	1.00000	
7	3424, 22, 3559	0.334	0.259	1.287	1.00000	
8	59, 3453, 50, 1224	-0.460	0.172	-2.680	0.10301	
9	121, 3449, 182, 130, 95, 425, 1693	0.679	0.567	1.197	1.00000	
10	291, 580, 398, 3535	1.318	0.298	4.430	0.00013	***
11	30, 39, 34	0.171	0.156	1.093	1.00000	
12	45, 3447, 924, 54	0.678	0.193	3.511	0.00626	**
13	188, 1533, 3469	0.228	0.208	1.095	1.00000	
14	1, 3434, 3446	-0.019	0.152	-0.128	1.00000	

[e] – The effect of phylogeny was tested using the model presented in Supplementary Tables 1 and 2. This post-hoc analysis compares an estimate for each phylogenetic cluster against the mean estimate for the whole dataset. *P*-values were calculated using two-sided Wald test and adjusted by Bonferroni method (*n*=14). The analysis was performed using R (version 3.5.1) and the package emmeans (version 1.3.0).

Supplementary Table 6.

Pairs of high and low evolvability parental strains selected for transcriptomic analysis. Pair 12 (ERR419045-ERR418493) was excluded from analysis due to batch effect

Pair	High evolvability strain				Low evolvability strain			
	Strain[f]	Survival	Cluster	MLST	Strain	Survival	Cluster	MLST
1	ERR418427	0.750	7	3424	ERR418568	0.083	7	22
2	ERR418483	1.000	6	101	ERR419071	0.000	2	109
3	ERR418500	1.000	13	188	ERR418539	0.000	13	188
4	ERR418522	0.917	3	97	ERR418938	0.000	3	26
5	ERR418530	0.750	5	8	ERR419229	0.000	5	8
6	ERR418583	1.000	1	12	ERR418597	0.083	1	12
7	ERR418609	1.000	11	30	ERR418998	0.000	11	39
8	ERR418658	1.000	12	45	ERR418719	0.000	12	45
9	ERR418857	0.750	6	5	ERR419074	0.000	6	5
10	ERR418968	1.000	8	3453	ERR419015	0.083	8	1224
11	ERR418997	1.000	5	8	ERR419022	0.000	5	8
12	ERR419045	0.833	1	12	ERR418493	0.000	1	12
13	ERR419052	1.000	14	1	ERR419241	0.083	14	3446
14	ERR419164	1.000	4	2255	ERR419138	0.000	4	15
15	ERR419186	1.000	8	59	ERR418706	0.000	8	Unknown

[f] – Strains are named using accession numbers of their whole-genome sequence reads available at Sequence Read Archive (www.ncbi.nlm.nih.gov/bioproject/PRJEB5261)

Supplementary Table 7.
Differential expression results for genes involved in the *S. aureus* SOS response. *P*-values were obtained using two-sided Wald test and adjusted by Benjamini-Hochberg method with the help of the DESeq2 package in R

Locus[g]	Gene name	Gene product	Two-sided Wald test	
			Log2 fold change	Adjusted *p*-value
SAR1986	SAR1986	ImpB/MucB/SamB family protein	0.495	0.15675376
SAR1377	SAR1377	ImpB/MucB/SamB family protein	-0.600	0.17991471
SAR1367	grlA	topoisomerase IV subunit A	-0.217	0.30857814
SAR1349	dinR	DNA damage-inducible repressor	-0.258	0.33455888
SAR1357	SAR1357	putative exonuclease	-0.342	0.39588223
SAR2261	SAR2261	putative membrane protein	-0.211	0.51914207
SAR1959	SAR1959	putative membrane protein	0.235	0.54014799
SAR1366	grlB	topoisomerase IV subunit B	-0.132	0.60258027
SAR0813	uvrA	excinuclease ABC subunit A	0.130	0.7054411
SAR1261	recA	recombinase A	-0.092	0.76493976
SAR1935	SAR1935	putative DNA repair exonuclease	-0.096	0.8053331
SAR2263	SAR2263	putative membrane protein	-0.088	0.84008921
SAR1356	SAR1356	putative exonuclease	0.079	0.87177237
SAR1597	SAR1597	putative DNA repair protein	0.046	0.90525486
SAR0812	uvrB	excinuclease ABC subunit B	0.041	0.92119027
SAR2262	SAR2262	putative UTP--glucose-1-phosphate uridylyltransferase	-0.016	0.96945147

[g] – loci correspond to annotation in MRSA252 genome (ASM1150v1).

Supplementary Table 8.

The effect of *norA* overexpression on ciprofloxacin resistance. Post-hoc analysis compares the estimates of half growth inhibition concentration (IC_{50}) among three strains (expression vector carrying *norA*, empty vector and no vector RN4220 strain) either (1) under the control of native promoter or (2) using *xyl/tetO* promoter and the induction with 5 ng μl^{-1} of anhydrotetracycline. *P*-values were adjusted using Bonferroni correction for multiple testing (*n*=9) [h]

Contrast	Estimate	Std. Error	Two-sided *t* test		
			t-value	*p*-value	
**Strain**					
empty pRMC2, induction vs. native	-0.007	0.011	-0.618	1.00000	
no vector, induction vs. native	-0.024	0.017	-1.395	1.00000	
pRMC2-norA, induction vs. native	0.077	0.023	3.399	0.01149	*
**Native promoter**					
empty pRMC2 vs. no vector	-0.046	0.015	-2.982	0.03404	*
empty pRMC2 vs. pRMC2-norA	-0.105	0.018	-5.769	1.26e-06	***
no vector vs. pRMC2-norA	-0.059	0.020	-2.963	0.03595	*
**Induction**					
empty pRMC2 vs. no vector	-0.029	0.013	-2.256	0.24094	
empty pRMC2 vs. pRMC2-norA	-0.188	0.017	-11.015	4.04e-17	***
no vector vs. norA	-0.159	0.019	-8.590	4.63e-12	***

[h] – IC_{50} was estimated by fitting a dose-response curve using 4-parameter log-logistic model. A curve for each strain/condition combination included 10 ciprofloxacin concentrations with 3 replicates per antibiotic concentration. The dose response analysis and post-hoc comparisons were performed using R package drc (version 3.0-1).

Supplementary Table 9.

The effect of *norA* overexpression on evolvability. The number of extinct vs. survived replicate populations are shown for 3 bacterial treatments (*norA* overexpression using vector pRMC2, empty vector pRMC2 control and no vector control). For a statistical test, see Supplementary Table 10

Strain	Survived	Extinct
empty pRMC2	12	28
no vector	9	31
pRMC2−norA	40	0

Supplementary Table 10.

The comparison of survival outcomes presented in Supplementary Table 9 using two-sided Fisher's exact test. Non-adjusted *p*-values are shown

Comparison	Fisher's exact test, *p*-value	
empty pRMC2 vs. pRMC2−norA	4e-12	***
no vector vs. pRMC2−norA	4e-14	***
empty pRMC2 vs. no vector	6e-01	

Supplementary Table 11.

The effect of efflux pump inhibition on ciprofloxacin resistance. The table compares half growth inhibition dose with ($IC_{50\ RES}$) or without (IC_{50}) 33 µM reserpine (chemical inhibitor of NorA). *P*-values were obtained using *t*-test. Non-adjusted *p*-values are shown [i]

Strain	IC_{50}	$IC_{50\ RES}$	$IC_{50}/IC_{50\ RES}$	Std. err	Two-sided t-test	
					t-value	*p*-value
**Low evolvability**						
ERR418562	0.032	0.029	1.159	1.490	0.107	0.91514
ERR418706	0.067	0.082	0.871	1.016	-0.127	0.89948
ERR418956	0.092	0.040	2.334	2.480	0.538	0.59223
ERR418982	0.064	0.082	0.643	1.605	-0.222	0.82471
ERR418996	0.109	0.113	0.936	0.270	-0.238	0.81256
ERR419040	0.112	0.092	1.241	0.215	1.122	0.26534
ERR419084	0.096	0.075	1.279	0.252	1.110	0.27042
ERR419203	0.114	0.127	0.900	0.088	-1.142	0.25703
ERR419241	0.303	0.116	2.605	0.525	3.059	0.00309
**High evolvability**						
ERR418500	0.332	0.244	1.369	0.085	4.340	0.00004
ERR418583	0.296	0.239	1.236	0.108	2.184	0.03215
ERR418609	0.171	0.125	1.366	0.154	2.374	0.02017
ERR418658	0.523	0.292	1.788	0.177	4.447	0.00003
ERR418997	0.094	0.090	1.048	0.146	0.330	0.74245
ERR419034	0.120	0.110	1.099	0.131	0.755	0.45246
ERR419047	0.157	0.254	0.620	0.085	-4.490	0.00003
ERR419052	0.474	0.287	1.653	0.139	4.707	0.00001
ERR419087	0.289	0.191	1.515	0.139	3.707	0.00040
**High evolvability (CC398)**						
ERR418467	0.143	0.202	0.707	0.101	-2.893	0.00501
ERR418476	0.212	0.132	1.608	0.391	1.557	0.12375
ERR418531	0.238	0.212	1.119	0.135	0.880	0.38170
ERR418607	0.301	0.262	1.150	0.084	1.793	0.07700
ERR418628	0.168	0.210	0.800	0.105	-1.905	0.06064
ERR418695	0.168	0.108	1.553	0.198	2.797	0.00658
ERR418970	0.245	0.132	1.855	1.523	0.561	0.57631
ERR419100	0.250	0.167	1.494	0.549	0.901	0.37057
ERR419111	0.202	0.139	1.453	0.181	2.498	0.01471

[i] – The IC_{50} and $IC_{50\ RES}$ estimates were computed by fitting a dose-response curve using 4-parameter log-logistic model using R package drc (version 3.0-1). The function EDcomp from the same package was used to test the relative difference $IC_{50}/IC_{50\ RES}$ for each strain

Supplementary Table 12.
The effect of efflux pump inhibition on evolvability. 16 replicate population for 27 strains were exposed to 1 mg l^{-1} ciprofloxacin for 5 serial transfers with or without efflux pump inhibitor (33 uM reserpine). The reported *p*-values were adjusted using Holm method (*n*=27)

Strain (SRA accession)	Populations survived out of 16		Fisher's exact test, *p*-value	
	Cipro	Cipro + Reserpine		
**Low evolvability**				
ERR418562	0	0	1.00000	
ERR418706	0	0	1.00000	
ERR418956	0	1	1.00000	
ERR418982	1	2	1.00000	
ERR418996	1	0	1.00000	
ERR419040	1	0	1.00000	
ERR419084	0	0	1.00000	
ERR419203	0	0	1.00000	
ERR419241	1	1	1.00000	
**High evolvability**				
ERR418500	15	3	0.00096	***
ERR418583	10	0	0.00521	**
ERR418609	10	0	0.00521	**
ERR418658	16	16	1.00000	
ERR418997	2	0	1.00000	
ERR419034	5	0	0.56396	
ERR419047	11	1	0.01270	*
ERR419052	16	9	0.10197	
ERR419087	10	1	0.03902	*
**High evolvability (CC398)**				
ERR418467	7	0	0.10197	
ERR418476	8	0	0.04160	*
ERR418531	16	3	0.00008	***
ERR418607	16	0	8.98e-08	***
ERR418628	5	0	0.56396	
ERR418695	4	0	1.00000	
ERR418970	8	0	0.04160	*
ERR419100	14	2	0.00117	**
ERR419111	10	0	0.00521	**

Supplementary Table 13.

The effect of efflux pump inhibitor on the relationship between ciprofloxacin resistance and evolvability. GLM includes intrinsic resistance and the presence of the inhibitor as predictors of evolvability. The analysis of deviance table is shown

	Df	Deviance	Resid. Df	Resid. Dev	Pr(>Chi)	
IC_{50}	1	299.221	52	296.891	< 2e-16	***
Reserpine	1	91.153	51	205.739	< 2e-16	***
IC_{50} × Reserpine	1	6.120	50	199.619	0.01337	*

Null deviance = 596.11, *d. f.* =53

Supplementary Table 14.

The effect of *norA* expression on the cell survival in the presence of ciprofloxacin (1 mg l^{-1}). Post-hoc analysis compares the number of cells at 0, 1, 2, 3, 4, and 5 hours after the exposure. Two-sided Wald tests were performed with the help of the R package emmeans and using Dunnett's contrasts (reference level = pRMC2-norA+cip). *P*-values were corrected by Bonferroni method (*n*=30) [j]

Contrast	Estimate	Std. Error	*d.f.*	*t* value	*p* value	
Time = 0 h						
RN4220+cip vs. pRMC2-norA+cip	-0.059	0.138	191	-0.431	1e+00	
empty pRMC2+cip vs. pRMC2-norA+cip	-0.040	0.132	191	-0.307	1e+00	
pRMC2-norA vs. pRMC2-norA+cip	-0.167	0.132	191	-1.271	9e-01	
pRMC2-norA+cip+res vs. pRMC2-norA+cip	-0.081	0.132	191	-0.617	1e+00	
pRMC2-norA+res vs. pRMC2-norA+cip	-0.137	0.132	191	-1.044	1e+00	
Time = 1 h						
RN4220+cip vs. pRMC2-norA+cip	-0.709	0.102	191	-6.930	2e-09	***
empty pRMC2+cip vs. pRMC2-norA+cip	-0.697	0.102	191	-6.826	3e-09	***
pRMC2-norA vs. pRMC2-norA+cip	0.517	0.102	191	5.064	3e-05	***
pRMC2-norA+cip+res vs. pRMC2-norA+cip	-0.799	0.102	191	-7.819	1e-11	***
pRMC2-norA+res vs. pRMC2-norA+cip	0.442	0.102	191	4.329	7e-04	***
Time = 2 h						
RN4220+cip vs. pRMC2-norA+cip	-0.816	0.091	191	-8.950	1e-14	***
empty pRMC2+cip vs. pRMC2-norA+cip	-0.782	0.091	191	-8.585	1e-13	***
pRMC2-norA vs. pRMC2-norA+cip	1.898	0.091	191	20.842	<1e-14	***
pRMC2-norA+cip+res vs. pRMC2-norA+cip	-0.754	0.091	191	-8.279	6e-13	***
pRMC2-norA+res vs. pRMC2-norA+cip	1.752	0.091	191	19.241	<1e-14	***
Time = 3 h						
RN4220+cip vs. pRMC2-norA+cip	-0.631	0.091	191	-6.928	2e-09	***
empty pRMC2+cip vs. pRMC2-norA+cip	-0.556	0.091	191	-6.108	2e-07	***
pRMC2-norA vs. pRMC2-norA+cip	3.582	0.091	191	39.336	<1e-14	***
pRMC2-norA+cip+res vs. pRMC2-norA+cip	-0.355	0.091	191	-3.901	4e-03	**
pRMC2-norA+res vs. pRMC2-norA+cip	3.392	0.091	191	37.252	<1e-14	***
Time = 4 h						
RN4220+cip vs. pRMC2-norA+cip	-0.404	0.102	191	-3.950	3e-03	**
empty pRMC2+cip vs. pRMC2-norA+cip	-0.283	0.102	191	-2.775	1e-01	
pRMC2-norA vs. pRMC2-norA+cip	5.177	0.102	191	50.676	<1e-14	***
pRMC2-norA+cip+res vs. pRMC2-norA+cip	-0.012	0.102	191	-0.117	1e+00	
pRMC2-norA+res vs. pRMC2-norA+cip	4.962	0.102	191	48.578	<1e-14	***
Time = 5 h						
RN4220+cip vs. pRMC2-norA+cip	-0.384	0.132	191	-2.923	8e-02	.
empty pRMC2+cip vs. pRMC2-norA+cip	-0.226	0.132	191	-1.722	7e-01	
pRMC2-norA vs. pRMC2-norA+cip	6.290	0.132	191	47.827	<1e-14	***
pRMC2-norA+cip+res vs. pRMC2-norA+cip	-0.133	0.132	191	-1.012	1e+00	
pRMC2-norA+res vs. pRMC2-norA+cip	6.063	0.132	191	46.100	<1e-14	***

[j] - Post-hoc analysis was based on a polynomial regression. Log-transformed population densities (shown on Figure 5a) were fitted using time variable (3 polynomial terms) and treatment as predictors.

Supplementary Table 15.
Oligonucleotides for cloning *norA* and sequencing resistant mutants

Name	Sequence 5'>3'	Description
ST398NM01_0772_fwd	taaaataagcttgatggtacTTGCAATTTGTCGTGGAAAAG	Cloning NorA using Gibson assembly
ST398NM01_0772_rev	ttgtaaaacgacggccagtgTTACAAATCTTGTTGTTTTAACTTAGCTC	Cloning NorA using Gibson assembly
TetR	GGCGAGTTTACGGGTTGTTA	Forward sequencing primer for pRMC2 upstream of the cloning site
pRS-marker	CGGCATCAGAGCAGATTGTA	Reverse sequencing primer for pRMC2 downstream of the cloning site
grlA1-R	ATCACTTCAGCTAAATTATGT	Amplification and sequencing *grlA* gene
glrA1-F	TGTTTTAGGTGATCGCTTTGG	Amplification and sequencing *grlA* gene
glrA2-F	ACATGGTCGCTATTAGTGATGG	Amplification and sequencing *grlA* gene
glrA2-R	ACGCGTTGTTTTAAATAGAGGCA	Amplification and sequencing *grlA* gene
gyrAseqF1	ACCAGTGAAATGCGTGAATCA	Amplification and sequencing *gyrA* gene
gyrAseqR1	TACCGCGATACCTGATGCAC	Amplification and sequencing *gyrA* gene
grlBseqF1	AGCTCGTGAAGATGCTCGTT	Amplification and sequencing *grlB* gene
grlBseqR1	TGGTCCTCTTGCATACCAAACT	Amplification and sequencing *grlB* gene
gyrBseqF1	ATCCACAAGTCGCACGTACA	Amplification and sequencing *gyrB* gene
gyrBseqR1	TATACAACGGTGGCTGTGCA	Amplification and sequencing *gyrB* gene

References for Supplementary Information

1 Fox, J. *Applied regression analysis and generalized linear models*. 3rd edn, (SAGE, Los Angeles, 2016).
