# Supplementary material for: Efflux pump activity potentiates the evolution of antibiotic resistance across S. aureus isolates
Source: Nat Commun. 2020 Aug 7;11:3970. doi: 10.1038/s41467-020-17735-y (PMC7414891; doi:10.1038/s41467-020-17735-y)
Supplement: Supplementary file 3 — Description of Additional Supplementary Files [file 41467_2020_17735_MOESM3_ESM.pdf]

Description of Additional Supplementary Files

File Name: Supplementary Data 1
Description: **List of strains,intrinsic resistance,evolvability.xlsx** Strains used in this study, their multilocus sequence type, survival in evolution experiment, and intrinsic resistance of parental strains

File Name: Supplementary Data 2
Description: **List of mutations identified in evolved strains.xlsx** Functional annotation of SNPs, indels and small structural variations identified in the evolved strains

File Name: Supplementary Data 3
Description: **Differentially expressed genes.xlsx** Genes identified as being significantly differentially expressed between high and low evolvability strains after 1.5 h of exposure to ciprofloxacin

File Name: Supplementary Data 4
Description: **Up- and down-regulated pathways.xlsx** Up- and downregulated KEGG (Kyoto Encyclopaedia of Genes and Genomes) pathways in high evolvability strains compared to low evolvability strains

File Name: Supplementary Data 5
Description: **GWAS results.xlsx** SNPs and their association with increased evolvability as determined by GWAS analysis
