# Supplementary material for: Efflux pump activity potentiates the evolution of antibiotic resistance across S. aureus isolates
Source: Nat Commun. 2020 Aug 7;11:3970. doi: 10.1038/s41467-020-17735-y (PMC7414891; doi:10.1038/s41467-020-17735-y)
Supplement: Supplementary file 9 — Reporting Summary [file 41467_2020_17735_MOESM9_ESM.pdf]

nature research

Corresponding author(s): Andrei Papkou

Last updated by author(s): Jul 9, 2020

Reporting Summary

Nature Research wishes to improve the reproducibility of the work that we publish. This form provides structure for consistency and transparency in reporting. For further information on Nature Research policies, see our Editorial Policies and the Editorial Policy Checklist.

Statistics

For all statistical analyses, confirm that the following items are present in the figure legend, table legend, main text, or Methods section.

n/a	Confirmed	
☐	☒	The exact sample size (n) for each experimental group/condition, given as a discrete number and unit of measurement
☐	☒	A statement on whether measurements were taken from distinct samples or whether the same sample was measured repeatedly
☐	☒	The statistical test(s) used AND whether they are one- or two-sided<br>*Only common tests should be described solely by name; describe more complex techniques in the Methods section.*
☐	☒	A description of all covariates tested
☐	☒	A description of any assumptions or corrections, such as tests of normality and adjustment for multiple comparisons
☐	☒	A full description of the statistical parameters including central tendency (e.g. means) or other basic estimates (e.g. regression coefficient) AND variation (e.g. standard deviation) or associated estimates of uncertainty (e.g. confidence intervals)
☐	☒	For null hypothesis testing, the test statistic (e.g. F, t, r) with confidence intervals, effect sizes, degrees of freedom and P value noted<br>*Give P values as exact values whenever suitable.*
☒	☐	For Bayesian analysis, information on the choice of priors and Markov chain Monte Carlo settings
☒	☐	For hierarchical and complex designs, identification of the appropriate level for tests and full reporting of outcomes
☒	☐	Estimates of effect sizes (e.g. Cohen's d, Pearson's r), indicating how they were calculated

Our web collection on statistics for biologists contains articles on many of the points above.

Software and code

Policy information about availability of computer code

Data collection	The optical density of bacterial cultures (to determine survival, resistance and growth rate) was recorded by a Synergy 2 plate reader (BioTek, USA) using Gen5 software ver.2.0 (BioTek).<br>Colony imaging (for counting colonies on agar plates) was performed on ColonyDoc-It 130 Imaging Station (UVP, Cambridge, UK) with the help of Doc-It Colony Counter software (UVP, Cambridge, UK).
Data analysis	The statistical analysis of results based on optical density data (population survival, dose-response and growth rate) was performed using R v3.5.1. In addition, the following R packages were used: drc v3.0-1 for dose-response analysis, pracma v2.2.2 for estimating the area under the curve (AUC), cluster v2.0.7-1 for non-hierarchical clustering and emmeans v1.3.0 for post-hoc comparisons.<br>Counting the number of colonies on agar plates was performed in ImageJ 1.51u using a custom script (available from gitlab https://gitlab.com/apson/sa_evolvability). The count data was exported into R v3.5.1 for statistical analysis. The R package rSalvador v1.7 was used for estimating mutation rates.<br>Whole-genome sequencing data was processed using the following software: Trimmomatic v0.36, Stampy v1.0.31, SAMtools v1.7, bcftools v1.7, BLAST v2.5.0, SPAdes v3.11.1, Prokka 1.12-beta, Snippy v4.0-dev2,breseq v0.33.1, Muscle v3.8.31, ISmapper v2.0.<br>RAxML v8.2.9, ClonalFrameML v.1.11, and PhyML v.3.3 were used for phylogenetic analysis.<br>The analysis of transcriptomics data was performed using Salmon v0.11.3, as well as the R packages DESeq2 v1.22.2 and limma v3.38.3.<br>bugwas 1.0 was used for GWAS analysis.<br>A Jupyter notebook showing the processing of next-generation sequencing data and an ImageJ script used for counting bacterial colonies are available from gitlab repository [https://gitlab.com/apson/sa_evolvability/]

For manuscripts utilizing custom algorithms or software that are central to the research but not yet described in published literature, software must be made available to editors and reviewers. We strongly encourage code deposition in a community repository (e.g. GitHub). See the Nature Research guidelines for submitting code & software for further information.

Data

Policy information about availability of data

All manuscripts must include a data availability statement. This statement should provide the following information, where applicable:

- Accession codes, unique identifiers, or web links for publicly available datasets
- A list of figures that have associated raw data
- A description of any restrictions on data availability

Whole-genome data for 222 parental strains from previous study are available from the European Nucleotide Archive, PRJEB5261 [https://www.ebi.ac.uk/ena/data/view/PRJEB5261]. Reference genome sequence of S. aureus MRSA252 is available from the GeneBank, BX571856.1 [https://www.ncbi.nlm.nih.gov/nuccore/BX571856.1]. Reference genome sequence of S. aureus JP080 is available from the GeneBank, [https://www.ncbi.nlm.nih.gov/nuccore/AP017922.1]. Whole-genome sequence data of 122 evolved strains were deposited at the Sequence Read Archive, BioProject PRJNA633882 [https://www.ncbi.nlm.nih.gov/bioproject/?term=PRJNA633882]. RNA sequencing data for 28 strains are available from Gene Expression Omnibus database, GSE150762 [https://www.ncbi.nlm.nih.gov/bioproject/?term=GSE150762]. Other data that support the findings of this study are available from figshare doi:10.6084/m9.figshare.c.4984364 [https://doi.org/10.6084/m9.figshare.c.4984364]. Source data are provided with this paper

Field-specific reporting

Please select the one below that is the best fit for your research. If you are not sure, read the appropriate sections before making your selection.

☒ Life sciences ☐ Behavioural & social sciences ☐ Ecological, evolutionary & environmental sciences

For a reference copy of the document with all sections, see nature.com/documents/nr-reporting-summary-flat.pdf

Life sciences study design

All studies must disclose on these points even when the disclosure is negative.

Sample size	We aimed to include a large and genetically diverse sample of S. aureus strains, representing diversity of this pathogen in the UK. The number and genetic representation of strains included in this study were limited by two factors: 1) the availability of strains sensitive to ciprofloxacin (many strains are already resistant); 2) the experimental capacity in terms of equipment, lab space and people involved. We had access to more than 400 strains sensitive to ciprofloxacin, out of which 260 were selected for experimental evolution. The sampling procedure was informed by whole-genome data (available for all strains from a previous study) in order to maximize diversity in a sample and avoid over-representation of genetically similar strains.
Data exclusions	38 out of 260 strain were excluded from the analysis, because the whole-genome sequences of their evolved populations did not match the expected sequences. Discordant samples were completely excluded from the analysis (including all the replicates from the same strain). These mismatches could have arisen due to either contamination during the experiment or due to the fact that some of the parental isolates were initially polyclonal (we have found some evidence of this). During experimental evolution only 2/6406=0.0003 control measurements had optical density indicating contamination (this number in the denominator includes OD measurements for all transfers). Data exclusion criteria were not pre-established
Replication	Experimental evolution of 222 strains was repeated twice. The first attempt failed due to experimental error. Experimental evolution with cell expressing norA and experiment evolution with norA inhibitor were performed one time. The transcriptome experiment and fluctuation test were performed one time. The determination of resistance to ciprofloxacin in 222 parental strains, in 83 evolved populations, in 27 strains with reserpine, and in 12 grlA mutants were performed one time. The experiment with norA overexpression in RN4220, the killing assay and estimation of growth rates were performed one time after smaller trial experiments. The results from the trial experiments were reproducible.
Randomization	The position of strains on 96-well plates during experimental evolution were randomized using a pipetting robot (the details are provided in Methods)
Blinding	Not applicable, because prior to the experimental evolution we did not have any expectation for a particular strain or a group of strains. In addition, all measurement were obtained using a plate reader (i.e. not subjected to human bias) using randomized design (see above).

Reporting for specific materials, systems and methods

We require information from authors about some types of materials, experimental systems and methods used in many studies. Here, indicate whether each material, system or method listed is relevant to your study. If you are not sure if a list item applies to your research, read the appropriate section before selecting a response.

Materials & experimental systems

n/a	Involved in the study
☒	☐ Antibodies
☒	☐ Eukaryotic cell lines
☒	☐ Palaeontology and archaeology
☒	☐ Animals and other organisms
☒	☐ Human research participants
☒	☐ Clinical data
☒	☐ Dual use research of concern

Methods

n/a	Involved in the study
☒	☐ ChIP-seq
☒	☐ Flow cytometry
☒	☐ MRI-based neuroimaging
